# Effects of continuous versus intermittent theta-burst TMS on fMRI connectivity

**DOI:** 10.3389/fnhum.2024.1380583

**Published:** 2024-05-31

**Authors:** Molly S. Hermiller

**Affiliations:** Department of Psychology, Florida State University, Tallahassee, FL, United States

**Keywords:** hippocampus, neuroplasticity, TMS, functional connectivity, theta-burst stimulation

## Abstract

Transcranial magnetic stimulation is a noninvasive technique that can be used to evoke distributed network-level effects. Previous work demonstrated that the Hippocampal-Cortical Network responds preferably (i.e., greater memory improvement and increases in hippocampal-network connectivity) to continuous theta-burst stimulation protocol relative to intermittent theta-burst and to 20-Hz rTMS. Here, these data were further analyzed to characterize effects of continuous versus intermittent theta-burst stimulation on network-level connectivity measures – as well as local connectedness – via resting-state fMRI. In contrast to theories that propose continuous and intermittent theta-burst cause local inhibitory versus excitatory effects, respectively, both protocols caused local decreases in fMRI connectivity around the stimulated parietal site. While iTBS caused decreases in connectivity across the hippocampal-cortical network, cTBS caused increases and decreases in connectivity across the network. cTBS had no effect on the parietal-cortical network, whereas iTBS caused decreases in the right parietal cortex (contralateral hemisphere to the stimulation target). These findings suggest that continuous theta-burst may have entrained the endogenous hippocampal-cortical network, whereas the intermittent train was unable to maintain entrainment that may have yielded the long-lasting effects measured in this study (i.e., within 20-min post-stimulation). Furthermore, these effects were specific to the hippocampal-cortical network, which has a putative endogenous functionally-relevant theta rhythm, and not to the parietal network. These results add to the growing body of evidence that suggests effects of theta-burst stimulation are not fully characterized by excitatory/inhibitory theories. Further work is required to understand local and network-level effects of noninvasive stimulation.

## 1 Introduction

Large-scale networks are often defined in human subjects as collections of discrete regions exhibiting temporally correlated activity, as measured by the blood-oxygen-level dependent signal in fMRI (i.e., “fMRI connectivity”). Functional properties of these networks can be probed noninvasively using transcranial magnetic stimulation (Fox et al., 2012), including frontal, occipital, and motor-cortical networks (Gratton et al., 2013; Rahnev et al., 2013; Mastropasqua et al., 2014; Valchev et al., 2015; Steel et al., 2016). The human hippocampal-cortical network (HCN) responds to repetitive stimulation delivered to parietal cortex (Eldaief et al., 2011; Halko et al., 2014), with long-lasting increases in fMRI connectivity throughout portions of the HCN following multi-day stimulation (Wang et al., 2014; Wang and Voss, 2015; Nilakantan et al., 2017; Kim et al., 2018; Warren et al., 2018; Freedberg et al., 2019; Warren et al., 2019; Hermiller et al., 2019b). These experiments have been important for causally testing the role of hippocampal-cortical networks in memory. However, mechanisms for network-level effects of rTMS are not fully understood. Some evidence suggests that certain rTMS protocols are capable of inducing trans-synaptic plasticity changes on functionally connected downstream regions (i.e., within a targeted brain network), modulating cognitive functions supported by these regions (Fox et al., 2012).

A previous study (Hermiller et al., 2019a, Hippocampus) evaluated the effectiveness of single sessions of different rTMS sequences at modulating HCN connectivity and memory retrieval. Specifically, this within-subjects design compared the effects of often used TMS protocols (e.g., continuous theta-burst, cTBS; intermittent theta-burst, iTBS; and 20-Hz rTMS) on memory-related hippocampal connectivity to other HCN regions, and found that the continuous theta-burst pattern caused the most robust increases in hippocampal connectivity and significantly enhanced memory retrieval (see Hermiller et al., 2019a, Hippocampus for details).

Notably, the theta-burst rhythm employed by the cTBS and iTBS protocols deliver bursts of gamma (50-Hz) triplets every 200-ms (5-Hz) in either continuous (cTBS) or intermittent (iTBS) trains (Huang et al., 2005; Rossi et al., 2009). TBS protocols were developed based on rodent and human studies indicating that theta rhythms are associated with long-term potentiation (LTP) (Berry and Thompson, 1978; Larson et al., 1986; Buzsaki, 2002). Lasting aftereffects on motor corticospinal output lasting between 30 and 60 min are generally reported for TBS protocols, with cTBS associated with inhibitory and iTBS associated with facilitatory cortico-motor effects (Pascual-Leone et al., 1994; Chen et al., 2003; Huang et al., 2005; Rossi et al., 2009; Di Lazzaro et al., 2011). However, effects of TBS in the motor cortex are not necessarily generalizable to other cortical areas. Notably, rTMS has been shown to evoke resonance-like in non-motor areas (Thut et al., 2011a,b; Chanes et al., 2013; Hanslmayr et al., 2014; Kim et al., 2016; Romei et al., 2016; Albouy et al., 2017; Lea-Carnall et al., 2017; Weinrich et al., 2017; Roberts et al., 2018; Riddle et al., 2019), including theta-patterned stimulation induced theta synchrony (Reinhart et al., 2015; Li et al., 2017). HCN regions exhibit neuronal synchrony and phase locking at theta-band frequencies (4-8-Hz) (Foster and Parvizi, 2012; Foster et al., 2013; Watrous et al., 2013; Foster et al., 2015), providing a possible mechanism for network communication and information processing (Buzsaki, 2002; Zhang and Jacobs, 2015). Theta-patterned stimulation of the rodent hippocampus preferentially induces long-term potentiation (Larson et al., 1986), which enhances connectivity of the hippocampal-cortical network (Canals et al., 2009). Therefore, theta-burst stimulation mimicking the “theta-nested gamma” activity pattern relevant to the HCN may optimally influence this network’s function via activity entrainment (i.e., resonance; see Thut et al. 2011a,b). Indeed, Hermiller et al. (2019a) found that the HCN responded preferentially to cTBS – but not iTBS or 20-Hz rTMS – leading to improved memory accuracy and increased memory-related hippocampal fMRI connectivity to other HCN regions. Here, we follow-up with this dataset to further evaluate the effects of continuous versus intermittent theta-burst stimulation on resting-state network connectedness, as well as local connectivity at the site of stimulation.

**Figure 1:**
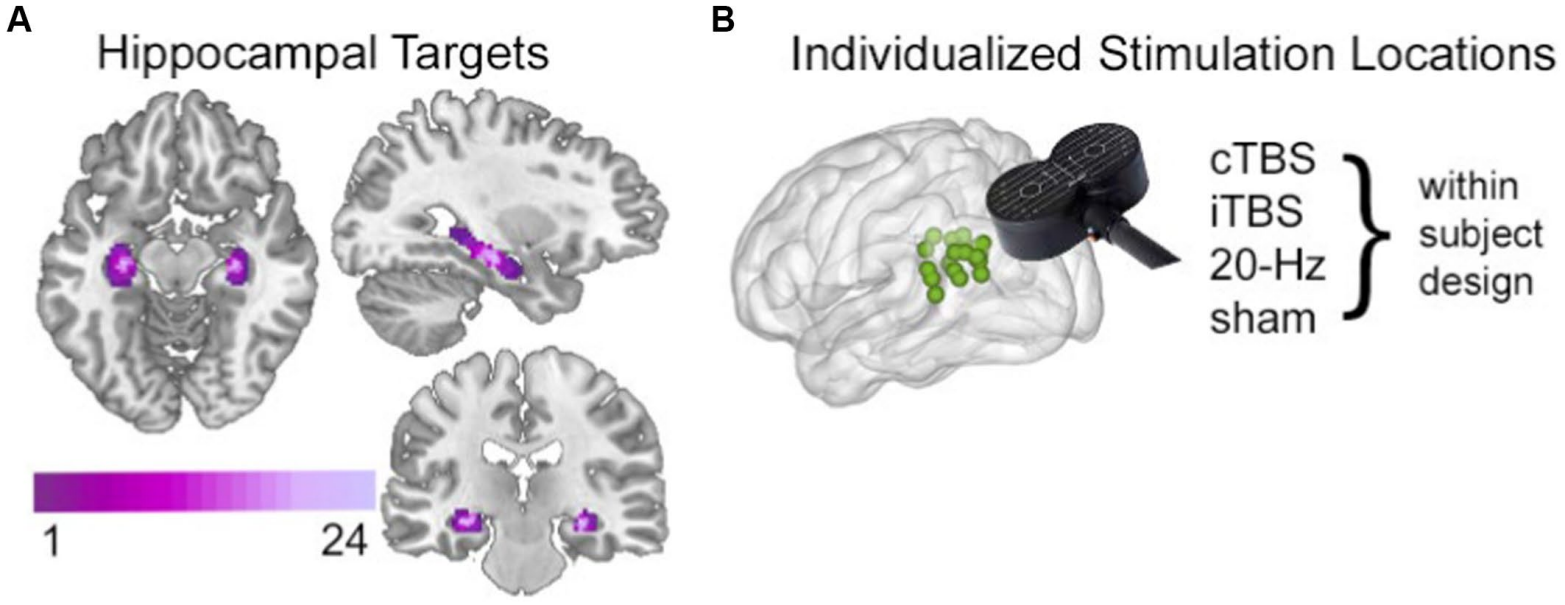
Targeting the HCN with noninvasive brain stimulation. **(A)** Hippocampal targets were defined as voxels with high fMRI interconnectivity within a bilateral anatomical mask of hippocampus. Targets are shown here as an overlap map across all subjects, with coloration indicating the number of subjects for which a given voxel was included as a hippocampal target. **(B)** The left parietal cortex stimulation locations were identified in each subject based on high fMRI connectivity with the subjectspecific hippocampal target, shown here as a green 2-mm sphere for each subject. A different stimulation condition was used in each experimental session in a within-subjects randomized counterbalanced order. The stimulation location was identical across the four conditions. Stimulation was delivered after the study phase and immediately before resting-state fMRI scanning and memory testing in each session. See Hermiller et al. (2019b), Hippocampus for full details.

As previously reported (Hermiller et al., 2019a), stimulation was delivered to a parietal cortex location of the HCN, defined in each subject based on maximal fMRI connectivity with bilateral hippocampus (Figure 1). Notably, fMRI connectivity of the HCN is consistent with its known anatomical organization (Squire and Zola-Morgan, 1991; Suzuki and Amaral, 1994; Vincent et al., 2006; Buckner et al., 2008). This includes projections from parietal cortex to hippocampus via retrosplenial and parahippocampal cortex (Mesulam et al., 1977; Mufson and Pandya, 1984) that presumably allow parietal stimulation to affect downstream HCN locations (Fox et al., 2012; Wang et al., 2014). Subjects received cTBS, iTBS, 20-Hz, rTMS, and sham stimulation targeting the same individualized parietal target in separate sessions over different (nonconsecutive) days. After each stimulation session, a resting-state fMRI scan was acquired. The effects of stimulation was quantified using resting-state fMRI network connectedness analysis (Cole et al., 2010; Gotts et al., 2012; Steel et al., 2016; Warren et al., 2018), which identified areas where stimulation significantly affected mean fMRI connectivity with other network voxels relative to sham stimulation condition.

## 2 Results

**Figure 2:**
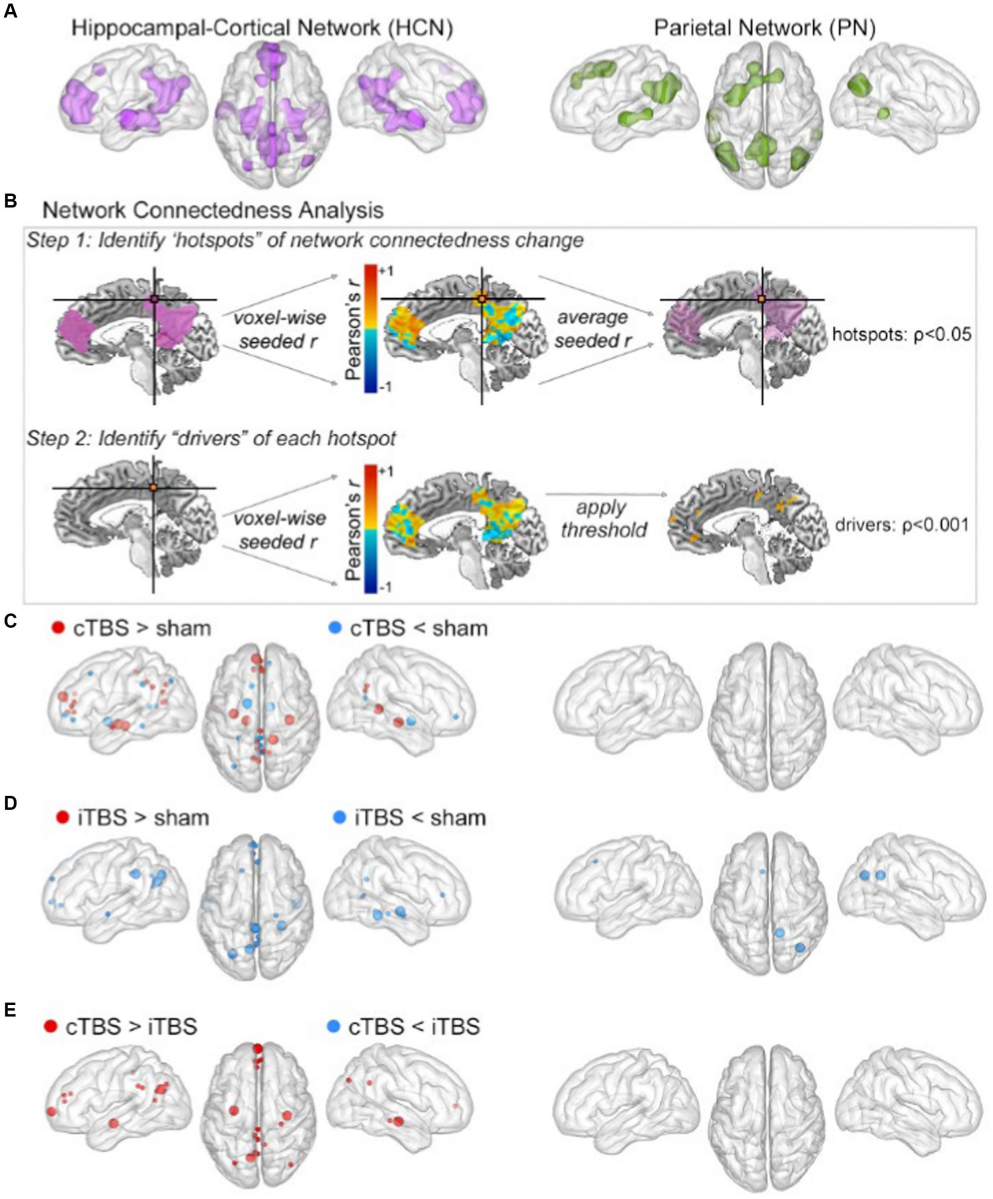
cTBS preferentially influenced HCN fMRI connectedness. **(A)** The HCN (left) and PN (right) masks were defined by resting-state fMRI connectivity with hippocampal and parietal targets, respectively. **(B)** Overview of the fMRI connectedness analyses performed in both networks. Step 1 identified hotspots of network connectedness changes (*p* < 0.05, cluster-size determined via Monte Carlo permutation). Step 2 used each hotspot in a network-constrained seed-based connectivity analysis to identify drivers of its connectivity change. **(C)** Clusters for cTBS versus sham shown as spheres at the center of mass in the HCN (left) and PN (right). Hotspots depicted as large spheres; drivers depicted as small spheres. Detailed cluster information is provided in Tables 1, 2. The same information for **(D)** iTBS versus sham and for **(E)** cTBS versus iTBS.

To evaluate the effects of theta-burst stimulation on network connectedness, we used a two-step voxel-wise measure of fMRI connectedness (Cole et al., 2010, Gotts et al., 2012, Steel et al., 2016, Warren et al., 2018) within two networks of interest: (1) the Hippocampal-Cortical Network (HCN), the brain regions with robust functional connectivity to the downstream hippocampus (i.e., our indirect target of our stimulation), and (2) the Parietal Network (PN), the brain regions with robust functional connectivity to the stimulated (i.e., local) parietal location. The HCN was defined at the group level as regions demonstrating high fMRI connectivity with the hippocampal targets identified for each subject using the sham condition fMRI data (Figure 2A, left). The PN was defined at the group level as regions demonstrating high fMRI connectivity with the parietal cortex stimulation location for each subject using fMRI data from the sham condition (Figure 2A, right).

The two-step fMRI connectedness analyses were performed as pairwise contrasts of stimulation conditions (i.e., cTBS vs. sham, iTBS vs. sham, cTBS vs. iTBS) within each network (i.e., HCN and PN). This analysis first identified the connectedness “hotspots,” which were contiguous voxels for which mean fMRI connectivity with all other network voxels differed significantly between stimulation conditions. Then, each hotspot was used as a seed in a seed-based connectivity analysis to identify the “drivers” of these mean whole-network connectivity effects, which were regions in the networks that significantly differed in connectivity with the hotspots due to stimulation condition (Figure 2B; see methods). Each pairwise comparison of stimulation conditions thus yielded a set of connectedness hotspots and their corresponding drivers in the HCN (Table 1) and PN (Table 2). Monte Carlo permutation testing was performed to determine appropriate cluster sizes to control for type-1 errors and multiple comparisons (see Methods).

**Table 1:** Numbered list of HCN connectedness clusters with their center Talairach coordinates, volume (mm^3^), peak standardized *t*-statistic (*z*), and approximate Brodmann Area (BA).

			x	y	z	mm^3^	*z*	BA
cTBS versus sham								
1	+	R Parahippocampal Gyrus	**−15**	**41**	**6**	**352**	**3.0**	**30**
2		L Paracentral Lobule	0	31	51	184	3.9	5
3		R Cingulate Gyrus	−10	56	27	256	4.3	31
4		L Precuneus	4	51	32	232	4.2	31
5		L Precuneus	2	67	35	208	4.1	7
6	−	L Parahippocampal Gyrus	**15**	**1**	**−8**	**344**	**2.0**	**28**
7		L Medial Frontal Gyrus	5	−51	0	896	3.3	10
8		R Medial Frontal Gyrus	−10	−48	−3	248	3.3	10
9		L Parahippocampal Gyrus	25	39	−13	408	3.3	37
10		L Cingulate Gyrus	4	40	35	264	3.3	31
11		L Posterior Cingulate	1	49	9	1,288	3.3	29
12	+	L Medial Frontal Gyrus	**5**	**−53**	**21**	**312**	**2.6**	**9**
13	+	L Parahippocampal Gyrus	**16**	**17**	**−14**	**304**	**3.4**	**35**
14		L Anterior Cingulate	8	−40	3	224	4.1	32
15		R Medial Frontal Gyrus	0	−50	17	1,464	4.7	9
16		L Middle Temporal Gyrus	54	4	−15	336	4.2	21
17		R Middle Temporal Gyrus	−58	16	−7	776	4.6	21
18		R Precuneus	−4	54	33	1,256	4.7	31
19	−	R Parahippocampal Gyrus	**−11**	**3**	**−7**	**272**	**2.0**	**28**
20		L Anterior Cingulate	8	−41	−6	200	3.3	10
21		L Medial Frontal Gyrus	1	−51	−0	432	3.3	10
22		L Superior Frontal Gyrus	9	−21	48	264	3.3	8
23		R Cingulate Gyrus	0	39	35	504	3.3	31
24		R Posterior Cingulate	−1	55	10	192	3.3	29
25		R Posterior Cingulate	−3	56	19	184	3.3	23
26		L Precuneus	31	72	39	184	3.3	19
27	+	R Hippocampus	**−34**	**18**	**−9**	**176**	**2.8**	**20**
28		L Anterior Cingulate	4	−48	3	168	4.0	32
29		L Anterior Cingulate	0	−40	16	368	4.5	32
30		L Posterior Cingulate	9	63	14	200	4.4	30
31	+	L Hippocampus	**31**	**11**	**−11**	**160**	**3.1**	**20**
32		R Anterior Cingulate	0	−43	10	1,648	5.0	32
33		R Medial Frontal Gyrus	−2	−39	24	168	3.9	9
44		L Cingulate Gyrus	4	44	38	168	4.3	31
35		L Precuneus	7	63	26	200	3.9	31
36		L Posterior Cingulate	3	65	16	216	4.4	31
iTBS versus sham								
1	−	R Cingulate Gyrus	**−1**	**40**	**36**	**1,360**	**2.0**	**31**
2		L Anterior Cingulate	2	−48	−1	192	3.3	32
3		R Parahippocampal Gyrus	−30	40	−8	208	3.3	36
4	−	R Fusiform Gyrus	**−31**	**37**	**−13**	**704**	**2.0**	**37**
5		L Superior Frontal Gyrus	5	−58	30	160	3.3	9
6		R Medial Frontal Gyrus	−1	−57	5	536	3.3	10
7		R Anterior Cingulate	−3	−38	11	344	3.3	32
8		L Middle Temporal Gyrus	55	6	−11	416	3.3	21
9		R Middle Temporal Gyrus	−55	8	−11	640	3.3	21
10		R Parahippocampal Gyrus	−25	25	−11	248	3.3	35
11		L Cingulate Gyrus	4	35	36	208	3.3	31
12		R Cingulate Gyrus	−4	50	29	1,200	3.3	31
13		R Posterior Cingulate	−6	54	10	304	3.3	30
14		R Precuneus	−1	60	21	240	3.3	23
15	−	L Precuneus	**26**	**70**	**35**	**392**	**2.0**	**19**
16		L Medial Frontal Gyrus	2	−60	4	496	3.3	10
17		L Superior Frontal Gyrus	15	−28	50	440	3.3	8
18		R Middle Temporal Gyrus	−53	7	−13	296	3.3	21
19		R Precuneus	−2	58	34	272	3.3	7
20	−	L Precuneus	**6**	**65**	**27**	**216**	**2.0**	**31**
21	−	R Superior Temporal Gyrus	**−49**	**12**	**−8**	**192**	**2.0**	**22**
cTBS versus iTBS								
1	**+**	L Hippocampus	**28**	**10**	**−14**	**544**	**3.6**	**34**
2		R Anterior Cingulate	−4	−49	8	1,448	4.4	10
3		L Medial Frontal Gyrus	1	−41	22	1,144	4.5	9
4		R Hippocampus	−29	26	−10	168	4.3	36
5		L Precuneus	0	32	47	344	4.3	5
6		R Cingulate Gyrus	0	44	29	464	4.0	31
7		R Precuneus	−8	50	34	592	4.7	31
8		L Precuneus	6	67	28	824	4.7	31
9		R Precuneus	−39	74	37	184	3.7	19
10	**+**	L Precuneus	**8**	**66**	**27**	**288**	**2.5**	**31**
11		L Cingulate Gyrus	4	39	31	296	4.7	31
12		L Posterior Cingulate	3	59	13	304	4.0	30
13		L Precuneus	27	72	33	336	4.6	19
14	**+**	L Medial Frontal Gyrus	**0**	**−63**	**1**	**264**	**2.8**	**10**
15		L Medial Frontal Gyrus	3	−48	20	832	4.2	9
16	**+**	R Hippocampus	**−34**	**16**	**−11**	**232**	**3.6**	**21**
17		L Medial Frontal Gyrus	0	−48	13	2016	4.8	10
18		R Cingulate Gyrus	0	61	25	1,592	4.3	31

Hotspots are in bold text and marked with their direction of change (-/+) in fMRI connectedness within the HCN.

**Table 2:** Numbered list of PN connectedness clusters with their center Talairach coordinates, volume (mm^3^), peak standardized *t*-statistic (*z*), and approximate Brodmann Area (BA).

			x	y	z	mm^3^	*z*	BA
cTBS versus sham								
		NONE						
iTBS versus sham								
**1**	**-**	R Angular Gyrus	**−37**	**68**	**30**	**480**	**2.0**	**39**
**2**	**-**	R Precuneus	**−14**	**51**	**30**	**264**	**2.0**	**31**
3		L Superior Frontal Gyrus	6	−23	48	232	3.3	8
4		R Declive (Cerebellum)	−18	75	−18	192	3.3	--
cTBS versus iTBS								
		NONE						

Hotspots are in bold text and marked with their direction of change (-/+) in fMRI connectedness within the PN.

### 2.1 Hippocampal-cortical network connectedness results

Results from the analysis of HCN connectedness for cTBS and iTBS (Figure 2) are presented in detail in Table 1. F or cTBS versus sham, seven hotspots with 29 corresponding driver regions were identified (36 total connectedness clusters; Table 1; Figure 2C, left), with a mix of increases and decreases in fMRI connectedness. These hotspots and drivers were distributed across the HCN, suggested widespread network-level effects in response to cTBS. Five hotspots were identified in the HCN fMRI connectedness analyses for iTBS relative to sham, producing a total of 21 connectedness clusters, all with decreases in HCN fMRI connectedness (Table 1; Figure 2D, left). In a direct comparison of cTBS to iTBS, four hotspots and 14 drivers showed significantly greater fMRI connectivity for cTBS (18 connectedness clusters; Table 1; Figure 2E, left). These clusters were located in core HCN regions, including bilateral hippocampus and medial and lateral prefrontal and parietal locations. This indicated substantially greater HCN engagement by cTBS than iTBS, particularly for core regions of the HCN such as the hippocampus and anterior and posterior midline areas.

### 2.2 Parietal network connectedness results

Results from the analysis of PN connectedness for cTBS and iTBS (Figure 2) are presented in detail in Table 2. cTBS did not differ from sham, failing to yield any hotspots or drivers (Figure 2C, right). Two hotspots and a total of four connectedness clusters were identified in the PN fMRI connectedness analyses for iTBS relative to sham (Table 2; Figure 2D, right). The hotspot in the right precuneus yielded drivers in the superior frontal gyrus and cerebellum, whereas the hotspot in the right angular gyrus did not yield drivers. Thus, the effects of iTBS on PN fMRI connectedness were mostly constrained within the lateral posterior PN regions. No hotspots or drivers were identified for cTBS relative to iTBS (Table 2; Figure 2E, right).

### 2.3 Local effects of stimulation on fMRI connectivity of parietal cortex

**Figure 3:**
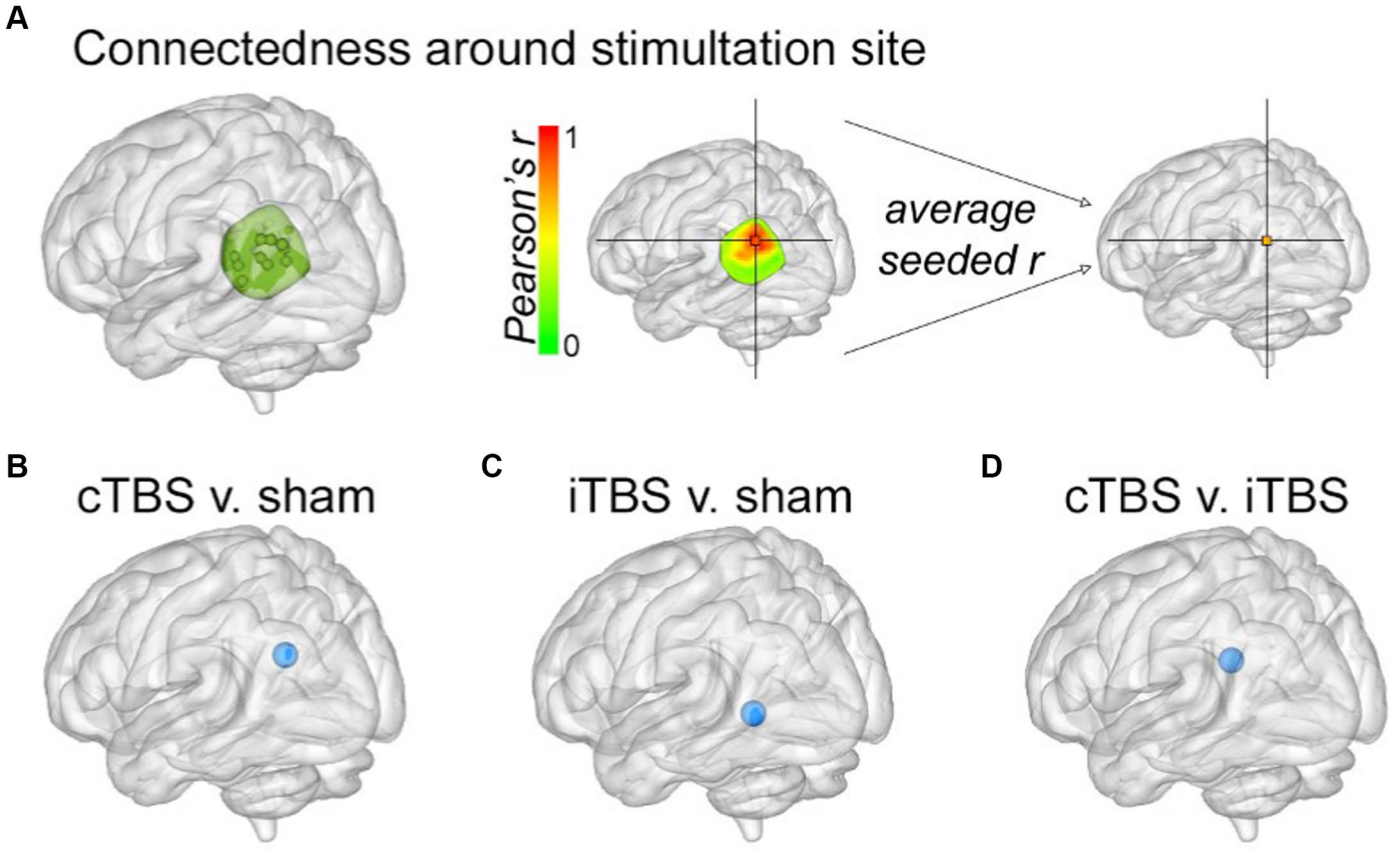
Local changes in parietal fMRI connectedness due to stimulation. **(A)** The parietal mask used when determining stimulation locations (see Methods in Hermiller et al., 2019a, Hippocampus) was used to measure changes in local fMRI connectedness. Results from the fMRI connectedness analyses within the parietal mask for **(B)** cTBS versus sham, **(C)** iTBS versus sham, and **(D)** cTBS versus iTBS. Blue spheres indicate decreased fMRI connectedness relative to sham for each of the indicated stimulation conditions, as in Figure 2.

In order to evaluate local effects of stimulation, we calculated fMRI connectedness within a mask of left parietal cortex that encompassed the stimulation locations and adjacent cortex (Figure 3A). Both theta-burst conditions caused clusters of significant connectedness decrease relative to sham (Figures 3B,C), with direct comparisons among theta-burst conditions indicating greater decreases for cTBS relative to iTBS (Figure 3D). It is notable that both cTBS and iTBS caused local decreases in connectedness but varied in their effects on network connectedness as described above. It is therefore unlikely that these difference network-level effects are due to differences in local effects of these stimulation conditions, as measured by fMRI connectedness.

## 3 Discussion

cTBS modulated HCN fMRI connectedness to a greater extent than did iTBS stimulation patterns. This continuous train of theta-patterned stimulation delivered to the parietal cortex influenced “core” HCN regions including hippocampus and parahippocampal cortex, changing their fMRI connectedness with other regions distributed throughout the HCN. In contrast, cTBS had minimal effects on the PN, which was defined as the fMRI network of the parietal cortex location that was directly stimulated. cTBS effects were thus observed downstream of the stimulation location, affecting fMRI connectivity of distributed HCN regions.

Mechanisms for network-level effects of TMS are not fully understood. Compensation-oriented explanations propose that networks “compensate” against changes in local activity due to stimulation, such that local excitation of regions with positive connections to a network will result in network connectivity reductions, and vice versa for local inhibition (Eldaief et al., 2011; Fox et al., 2012; Cocchi et al., 2015; Steel et al., 2016; Davis et al., 2017). Often, cTBS is considered an inhibitory sequence and iTBS is considered excitatory, based primarily on cortico-motor effects (Pascual-Leone et al., 1994; Chen et al., 2003; Huang et al., 2005). The parietal area stimulated in this study has known positive connectivity with the HCN. Consistent with compensation explanations, we found decreases in downstream connectivity in the HCN due to iTBS relative to sham. However, the effects of cTBS were more complicated, with both positive and negative downstream effects relative to sham. Furthermore, the effects of cTBS were specific to the HCN, with no significant changes identified in the PN. iTBS caused decreases in the right parietal region of the PN (contralateral side of the stimulation target). Thus, cTBS and iTBS did not produce opposite effects on HCN or PN fMRI connectivity, as would have been expected based on compensation-oriented explanations of network-level TMS effects due to putative inhibitory/excitatory local effects. Rather, these effects may instead be indicative of a network-level response to the resonance of the stimulation pattern. That is, intermittent trains of theta-burst did not entrain the putative endogenous HCN theta rhythm as effectively as the continuous train, and thus, did not induce as robust transient aftereffects on hippocampal-cortical network connectivity.

Resonance-oriented explanations propose that stimulation applied at frequencies matching the targeted network’s endogenous activity rhythms entrains oscillatory neural activity throughout network regions and promotes network synchrony and related functions (Thut et al., 2011a,b; Chanes et al., 2013; Kim et al., 2016; Romei et al., 2016; Salinas et al., 2016; Lea-Carnall et al., 2017; Weinrich et al., 2017). Indeed, resonance-like effects have been demonstrated by findings that noninvasive theta-patterned stimulation synchronizes theta oscillations (Reinhart et al., 2015; Li et al., 2017). Human hippocampal theta-frequency activity and theta phase locking with other HCN regions have been associated with working and long-term memory functions (Goutagny et al., 2009; Axmacher et al., 2010; Steinvorth et al., 2010; Foster and Parvizi, 2012; Lega et al., 2012; Foster et al., 2013; Watrous et al., 2013; Kaplan et al., 2014; Foster et al., 2015; Lin et al., 2017). Theta synchrony among HCN regions may support information processing and interregional communication (Buzsaki, 2002; Buzsaki and Draguhn, 2004; Burke et al., 2013; Zhang and Jacobs, 2015). The frequency-specificity of these effects support our hypothesis that the HCN would be preferentially engaged by stimulation continuously engaging its endogenous theta rhythm (Buzsaki, 2002; Anderson et al., 2010; Foster and Parvizi, 2012; Lega et al., 2012; Foster et al., 2013; Burke et al., 2014; Zhang and Jacobs, 2015; Lega et al., 2017; Lin et al., 2017). This may also explain in part why the PN (i.e., a distributed fronto-parietal network) displays beta-frequency activity as its hallmark (Rosanova et al., 2009; Samaha et al., 2017; Riddle et al., 2019). A limitation of the current study is that interregional communication was measured only indirectly as fMRI connectivity, which is a metric of low-frequency coupling of the fMRI signal at frequencies below the theta band (Foster et al., 2015; Hacker et al., 2017), although it is possible that theta-frequency neural activities contribute to fMRI connectivity measures via vasomotor entrainment (Mateo et al., 2017).

In line with the theory that theta-burst stimulation may cause downstream HCN entrainment, we conducted a simultaneous TMS/MRI study and delivered 2-s volleys of theta-burst stimulation to individualized parietal locations of subject’s HCN. We found site-specific (i.e., HCN vs. out-of-network location) and frequency-specific (i.e., theta-burst vs. 12.5-Hz) effects of stimulation, such that theta-burst delivered to the HCN improved memory performance and increased hippocampal BOLD signal during encoding (Hermiller et al., 2020). Notably, the 2-s volleys of theta-burst employed here was not an intermittent train of TBS (2-s on, 8-s off). Rather, the 2-s volleys were randomly interleaved with a range of ~11–39 s between volleys. Thus, the volleys were unlikely to have cumulative effects throughout the session as would a train of iTBS (Huang et al., 2005; Demeter et al., 2016). Instead, the 2-s volleys served to immediately affect activity in the hippocampus at an immediate trial-level, putatively via entrainment with the endogenous HCN theta rhythms. Indeed even single stimulation pulses have been reported to evoke activity at the natural frequencies of brain networks, with distinct evoked activity frequencies for various cortical regions (Rosanova et al., 2009). The immediate effects of stimulation reported by Hermiller et al. (2020) suggest that it selectively influenced hippocampal neural activity, as opposed to neuroplasticity and/or neuromodulatory mechanisms that can support persistent/long-lasting effects of stimulation on network function (Cirillo et al., 2017). It is possible that brief TBS volleys affect activity in only those areas most sensitive to this stimulation pattern (e.g., hippocampus) whereas more extensive network-wide effects are recruited with longer theta-burst trains (see Hermiller et al., 2019a, 2022). Further study, including directly measuring hippocampal theta activity as a result of theta-burst stimulation, will be required in order to directly test this hypothesis.

It is noteworthy that intermittent versus continuous theta-burst varied in their effects on the distributed networks (HCN vs. PN), but caused similar local effects at the site of stimulation. That is, both theta-burst conditions decreased fMRI connectedness around the stimulated location (Figure 3). These results indicate that local effects of theta-burst stimulation may not be fully characterized as either inhibitory or excitatory, and that local disruption may fundamentally differ how distributed network-level effects are achieved. Furthermore, although stimulation was delivered to a parietal cortical location, the most robust effects were on fMRI connectedness of the downstream hippocampus and its network locations, rather than within the network of regions with robust connectivity to the stimulated parietal site.

To summarize, these findings demonstrate that distinct large-scale brain networks (i.e., HCN vs. PN) are differentially engaged by network-specific noninvasive stimulation delivered to the same location (i.e., individualized parietal locations). The current findings add to this evolving understanding of network-level neuromodulation by suggesting that correspondence between stimulation and endogenous network rhythms could be an important factor in the network’s response to stimulation. Indeed, it was particularly noteworthy that the network-level effects that varied based on stimulation continuity in the current study, as local effects showed little variation. Furthermore, the network-level effects were specific to the network (i.e., HCN rather than PN) that reportedly has intrinsic rhythms that may have been entrained to the theta-burst stimulation pattern. This is a striking demonstration that networks stimulated location may respond preferentially to specific rhythms, and is at odds with existing compensation-oriented explanations of local stimulation effects on networks, which suggest that network-level changes are due to a “balancing” against purely local excitation or inhibition of activity by stimulation (Eldaief et al., 2011; Fox et al., 2012; Cocchi et al., 2015; Steel et al., 2016).

## 4 Methods

### 4.1 Participants

A final sample of 24 participants with complete datasets was included in all analyses (14 females, ages 19–28 years, average age = 23.5, SD = 2.6). The target sample size of 24 was based solely on previous studies using similar methods (Eldaief et al., 2011; Nee and D’Esposito, 2016). All participants gave written informed consent approved by the Northwestern University Institutional Review Board and were paid for participation. See Hermiller et al. (2019a), Hippocampus for further details about recruitment, eligibility, and exclusion criteria.

### 4.2 Experimental procedure

All experiment procedures are reported in detail in Hermiller et al. (2019a), Hippocampus. In sum, participants completed a baseline session and four experimental sessions on separate days using a within-subjects, single-blinded, counterbalanced design. The experimental sessions were separated by at least 48 h, with an average interval of 3.6 days between each session (range 2.0–5.8 days). Each experimental session consisted of a short practice session, during which participants ran through an example of the different phases of the day’s experiment and verbally confirmed they understood task instructions. After this, the experimental session commenced, starting with a memory task study phase, followed by TMS, resting-state fMRI scanning, and a memory task test phase. The memory task and results are not reported here; please see Hermiller et al. (2019a), Hippocampus for details about the memory task and the effects of stimulation on memory performance and memory-related connectivity.

### 4.3 Transcranial magnetic stimulation

A MagPro X100 stimulator and a MagPro Cool-B65 liquid-cooled butterfly coil (MagVenture A/S, Farum, Denmark) were used to deliver stimulation, along with an MRI-guided navigation system using individual MRIs (Localite GmbH, St. Augustin, Germany) to ensure accurate and reproducible targeting. Participants sat in an ergonomic chair with a stabilizing headrest and a vacuum-conforming pillow. Resting motor threshold (RMT), the minimum percentage of stimulator output (%SO) needed to produce visible contractions of the right *abductor pollicis brevis* (i.e., thumb muscle) for five out of 10 consecutive pulses, was determined during each subject’s baseline session. As previously reported, RMT values ranged between 34 and 68 %SO (mean = 49.2 %SO, SD = 7.5).

A different stimulation condition (cTBS, iTBS, 20-Hz rTMS, or sham) was delivered in each of the four different sessions/days, in a counterbalanced order across subjects (see Hermiller et al., 2019a, Hippocampus for details). Theta-burst stimulation utilized in the reported datasets here followed standard guidelines (Huang et al., 2005; Rossi et al., 2009, 2021), with a total of 600 pulses arranged in 50-Hz triplet bursts delivered every 200-ms (5-Hz). In cTBS, bursts were delivered in one continuous train (40-s duration), whereas in iTBS, bursts were delivered in intermittent trains (2-s on, 8-s off; 190-s duration). Theta-bust stimulation was delivered at an intensity of 80% RMT. The sham condition used a standard 20-Hz rTMS protocol (2-s on, 28-s off; 20 min duration), but with intensity lowered to 10% RMT as to be unlikely to affect neuronal activity. A 20-Hz active condition was also utilized, but not reported here.

TMS was delivered in a 20 min protocol while participants played “2048,” a single-player sliding-block puzzle on a computer tablet.^1^ This game served to engage the participants during the 20-min stimulation protocol, to prevent them from rehearsing the learned stimuli from the encoding phase, and to distract them from the variances in stimulation patterns across sessions. We chose this game as it includes numbers (i.e., no overlapping stimuli or semantics with the memory task stimuli), requires low motor effort (i.e., finger swipe) and is not timed (i.e., so participants would not be. jumpy’ or on edge to make fast responses, which could potentially cause head movement during the TMS delivery). TMS was delivered in a room adjacent to the MRI scanner room. Stimulation effects have been reported to last approximately 60 min post-stimulation (Huang et al., 2005; Rossi et al., 2009; Thut and Pascual-Leone, 2010; Wischnewski and Schutter, 2015). All subsequent testing procedures occurred within this time window.

### 4.4 MRI acquisition and preprocessing

See Hermiller et al. (2019a), Hippocampus for full details on MRI data collection and preprocessing. In summary, a Siemens 3 T Prisma whole-body scanner with a 64-channel head coil was used to collect resting-state functional images (TE 22-ms; TR 555-ms; flip angle 47°; voxel resolution 2.0×2.0×2.0-mm, 550 frames, 64 slices, multi-band factor of 8). Participants were instructed to lie as still as possible, to keep their eyes open and focused on a fixation cross presented in the center of the screen, and to let their minds wander without thinking of anything specific. Structural images were acquired using a T1-weighted MPRAGE sequence (TE 1.69-ms; TR 2170-ms; TI 1100-ms; flip angle 7°; voxel resolution 1.0×1.0×1.0-mm, 176 frames). MRI data were preprocessed using AFNI software (Cox, 1996), and included the outlier suppression, spatial normalization to the Talairach et al. (1988) Colin27 template, spatial smoothing (4-mm Gaussian smoothing kernel), and signal intensity normalization. Bandpass filtering (0.01–0.1-Hz), motion censoring (values replaced with zero), and nuisance time series (estimates of motion parameters and derivatives) were detrended from each voxel simultaneously as a linear regression model to yield a residual time series for subsequent analysis reported here.

### 4.5 Individualized stimulation target identification

Individualized hippocampal targets and parietal cortex stimulation locations were defined for each subject via fMRI connectivity measures using the data collected during each subject’s baseline session (Figure 1). Additional details are provided in Hermiller et al. (2019a), Hippocampus.

### 4.6 Network definition

The HCN and the PN were defined based on the individualized hippocampal targets and stimulation locations, respectively. These served as seeds in a group-level voxel-wise whole-brain connectivity analysis using the sham fMRI datasets. Contiguous voxels (*3dClustSim* cluster size threshold α < 0.01) with significant (*p* < 1.0×10^−8^) functional connectivity with the target were saved as network regions, and dilated (*3dmask_tool*). All surviving voxels were saved as the network mask. The HCN mask consisted of 15,325 voxels and the PN mask consisted of 9,047 voxels, with 2,833 voxels overlapping between networks (Figure 2A).

### 4.7 Statistical analyses methods

Statistical significance between each stimulation condition relative to sham was evaluated at the group-level using paired two-tailed *t*-tests. The sham condition was used in this comparison (i.e., rather than the baseline resting-state scan), as the sham condition underwent the exact same experimental features (e.g., encoding phase of the memory task, 2048 gam during stimulation session). The baseline resting state scan was not preceded with these features. Separate results were obtained for each stimulation contrast and were not used in analyses of other stimulation contrasts, thus eliminating circular analysis. Imaging analysis was done using AFNI (Cox, 1996) and Matlab (The MathWorks, Inc. Natick, MA, United States).^2^ MRI results were visualized using the BrainNet Viewer Matlab toolbox (Xia et al., 2013).

### 4.8 fMRI network connectedness analyses

A two-step voxel-wise within-network analysis was performed to identify voxels within networks of interest that changed in fMRI connectedness with other voxels within the network due to stimulation (Cole et al., 2010; Gotts et al., 2012; Steel et al., 2016; Warren et al., 2018). This yielded a set of connectedness clusters (“hotspots” of network connectedness change) (see Figure 2B, top). These hotspots were then used as seeds in seed-based connectivity analyses (constrained to the network the hotspot was identify in) to identify the “drivers” of each hotspot’s connectivity change (see Figure 2B, bottom). This was performed separately for each stimulation condition contrast in each network (Tables 1, 2; Figures 2C–E).

Step 1 (determine “hotspots”): Network fMRI connectedness maps were created by correlating (Pearson’s *r*) the time series in each voxel with every other voxel in the network-of-interest mask, averaging the correlations, and assigning the mean value to that voxel in the subject’s network connectedness map (*3dTcorrMap*). Fisher’s *z* transformation was applied to yield normally distributed network connectedness maps for each subject in each stimulation condition. Differences in network connectedness between stimulation conditions were evaluated at the group-level to identify hotspots of network connectedness change using *post-hoc* paired *t-*tests (two-tailed) in each voxel (*3dttest++*) to identify clusters of voxels with differences in network connectedness (*p* < 0.05 uncorrected, *z(t)*-threshold = 1.96). A statistical threshold of *p* < 0.05 was used to identify these hotspots (Gotts et al., 2012; Steel et al., 2016), considering the dilution of correlation strength that occurs when averaging connectedness across the entire network. The rationale for using a lenient threshold in this first step, was to be as inclusive as possible when determining hotspots of change. Monte Carlo permutation testing was performed to determine the appropriate hotspot cluster-size threshold in each network and to reduce the number of type 1 errors. The *t-*tests were repeated 100 times for each stimulation condition relative to sham in each network, with random flipping of condition labels for half the subjects to generate a probability distribution of hotspot sizes. A *α* < 0.05 cutoff yielded a hotspot cluster-size threshold of 20 voxels on the HCN distribution and 28 voxels on the PN distribution. Using these permutation-defined cluster-size thresholds, a set of hotspots was determined for each stimulation condition contrast in each network (Tables 1, 2). Subsequent analyses with a set of hotspots were performed using datasets only from the two stimulation conditions that were contrasted to determine that set of hotspots.

*Step 2: Identify “driver” regions of the hotspots*. The spatially averaged time series of each hotspot was used as a seed in seed-based, voxel-wise analyses (*3dTcorr1D*). A Fisher’s *z* transformation was applied to yield a normally distributed network correlation map for the hotspot for each subject in the relevant stimulation conditions. Between-condition differences in a hotspot’s connectivity were evaluated at the group level to identify drivers of that hotspot’s connectivity change. Monte Carlo permutation testing was performed to determine the appropriate driver cluster-size threshold for each set of hotspots. The difference in connectivity for each hotspot was evaluated using *post-hoc* paired *t-*tests (two-tailed) in each voxel (*3dttest++*) to identify clusters of voxels with differences in hotspot connectivity (*p* < 0.001 uncorrected, *z(t)*-threshold = 3.291). This was repeated 100 times for each hotspot, with random flipping of condition labels for half the subjects to generate a probability distribution of driver sizes. A *α* < 0.05 cutoff was used to determine the driver cluster-size threshold for each set of hotspots. Across both networks and all sets of hotspots, the number of voxels required to meet a *α* < 0.05 cutoff ranged from 7 to 15. A more conservative 20+ voxel cluster-size threshold was imposed to determine the drivers across both networks.

Each set of stimulation condition contrasts generated separate lists of connectedness clusters (hotspots and drivers) in each network. The maximum standardized *t*-statistic (*z*-score) was extracted (*3dROIstats*) for each connectedness cluster (Tables 1, 2).

## Data availability statement

Publicly available datasets were analyzed in this study. This data can be found at: https://nda.nih.gov/.

## Ethics statement

The studies involving humans were approved by Northwestern University Institutional Review Board. The studies were conducted in accordance with the local legislation and institutional requirements. The participants provided their written informed consent to participate in this study.

## Author contributions

MH: Data curation, Formal analysis, Visualization, Writing – original draft, Writing – review & editing.

## Footnotes

^1^ https://gabrielecirulli.github.io/2048/

^2^ http://www.mathworks.com
